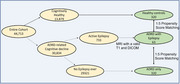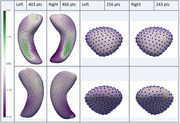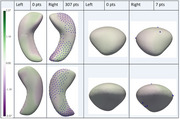# Hippocampus and Amygdala Volume and Morphology in Neurodegenerative Disorders with Co‐morbid Epilepsy

**DOI:** 10.1002/alz70857_097534

**Published:** 2025-12-24

**Authors:** Ifrah Zawar, Shen ZM Zhu, Jaideep Kapur, Mark S Quigg, Carol A Manning, P Thomas ZM Fletchet

**Affiliations:** ^1^ University of Virginia, Charlottesville, VA, USA

## Abstract

**Background:**

Epilepsy is common in dementia. However, the neuroimaging correlates of epilepsy in AD and non‐AD dementias remain unexplored. We investigated mesial temporal morphology and volumes in AD (AD+Epi) and non‐AD dementias (non‐AD+Epi) with epilepsy.

**Method:**

Using multicenter data from 39 US Alzheimer's disease centers (9/2005–12/2021), participants were classified into Group 1 (dementia with epilepsy: AD+Epi, nonAD+Epi); Group 2 (dementia without epilepsy: AD‐Epi, non‐AD‐Epi); and Group 3 (healthy controls, HC). Group 1 participants with available MRI scans were matched to Groups 2 and 3 (Figure 1) using fixed‐ratio, optimal propensity score matching by age, sex, and dementia type (AD vs nonAD).

Hippocampal and amygdalar segmentation from MRI was performed using FreeSurfer. Point distribution models created via ShapeWorks quantified morphological differences in the left and right hippocampi (512 points) and amygdalae (256 points). The volume of structure/Total intracranial volume yielded normalized volumes for hippocampi and amygdalae. Multivariate analysis of covariates (MANCOVA), adjusted for age, sex, intracranial volume, and dementia severity, identified statistically significant local morphological and normalized volume group differences. We compared AD+Epi vs AD‐Epi, AD+Epi vs HC, AD‐Epi vs HC, nonAD+Epi vs nonAD‐Epi, nonAD+Epi vs HC and nonAD‐Epi vs HC.

**Result:**

Of 703 included participants (average age:70.78 years, 391 (55.62%) female), 35 had AD+Epi, 28 nonAD+Epi, 183 AD‐Epi, 137 nonAD‐Epi, and 320 HC. AD‐Epi and NonAD‐Epi exhibited uniform hippocampal and amygdalar morphological atrophy bilaterally. In contrast, AD+Epi demonstrated morphological atrophy in the hippocampal bodies and tails bilaterally with sparing of the hippocampal heads, more pronounced inward deviations on mesial and lateral surfaces, and outward deviations in the middle hippocampal body bilaterally on the superior surface (Figure 2). NonAD+Epi showed significant morphological atrophy in the right hippocampal head, tail, and amygdala (Figure 3). No group volume differences were found except for smaller left hippocampal volumes in AD‐Epi than in HC.

**Conclusion:**

We identified hippocampal body and tail atrophy in AD+Epi and right hippocampal head, tail, and amygdalar atrophy in nonAD+Epi demonstrating that mesial temporal morphology may serve as a neuroimaging correlate for epilepsy in ADRD. These lateralized and region‐specific patterns highlight the possible role of epilepsy in altering the trajectory of neurodegeneration in ADRD, which warrants further investigation.